# Understanding the Influence of Rheology on Biofilm Adhesion and Implication for Food Safety

**DOI:** 10.1155/ijfo/2208472

**Published:** 2024-12-31

**Authors:** Adedola Adeboye, Helen Onyeaka, Zainab Al-Sharify, Nnabueze Nnaji

**Affiliations:** ^1^African Food Research Network, Pretoria 0002, Gauteng, South Africa; ^2^School of Chemical Engineering, University of Birmingham, Edgbaston, Birmingham B152TT, UK; ^3^Pharmacy Department, Al Hikma University College, Baghdad, Iraq; ^4^Department of Environmental Engineering, College of Engineering, Al-Mustansiriyah University, Baghdad, Iraq

**Keywords:** biofilm, fluid flow, food industry, non-Newtonian fluids, shear stress, viscosity

## Abstract

Understanding biofilm rheology is crucial for industrial and domestic food safety practices. This comprehensive review addresses the knowledge gap on the rheology of biofilm. Specifically, the review explores the influence of fluid flow, shear stress, and substrate properties on the initiation, structure, and functionality of biofilms, as essential implications for food safety. The viscosity and shear-thinning characteristics of non-Newtonian fluids may impact the attachment and detachment dynamics of biofilms, influencing their stability and resilience under different flow conditions. The discussion spans multiple facets, including the role of extracellular polymeric substances (EPSs) in biofilm formation, the impact of rheological attributes of biofilm on their adhesion to surfaces, and the influence of shear forces between biofilms and substrate's surface characteristics on biofilm stability. Analytical techniques, encompassing rheometry, microscopy, and molecular biology approaches, are scrutinized for their contributions to understanding these interactions. The paper delves into the implications for the food industry, highlighting potential risks associated with biofilm formation and proposing strategies for effective control and prevention. Future research directions and the integration of rheological considerations into food safety regulations are underscored as pivotal steps in mitigating biofilm-related risks. The synthesis of microbiology, materials science, and engineering perspectives offers a multidimensional exploration of rheology–biofilm interactions, laying the groundwork for informed interventions in diverse industrial settings.

## 1. Introduction

Microorganisms known as foodborne pathogens can make people sick when they eat foods contaminated with foodborne pathogens. Foodborne pathogens seriously jeopardize public health and food safety everywhere [1]. It is essential to comprehend the importance of foodborne pathogens in order to put preventative measures into action and guarantee the security of the food supply chain. The risk of contamination and the spread of foodborne pathogens may be decreased by taking steps like maintaining excellent hygiene, preparing food at the right temperature, preventing cross-contamination, and routinely testing and monitoring food items. To guarantee food safety and avoid cross-contamination, the food and beverage sector strictly enforces cleanliness standards and procedures in its manufacturing facilities [2]. Nevertheless, biofilms create conditions that often undermine the effectiveness of food safety precautions like washing and disinfection, putting food handlers and customers at risk of hazards that are “hidden in plain sight.” According to Srey, Jahid, and Ha [3] and Achinas, Charalampogiannis, and Euverink [2], biofilms are organized colonies of bacteria that stick to surfaces and create a protective matrix. They are common in pipelines, packing materials, and equipment used in a variety of food processing and storage settings [3, 4]. Biofilm generation has a variety of effects on settings used in food processing and storage. Food items can get contaminated, and the risk of foodborne diseases can rise due to the role that biofilms play as reservoirs for pathogens and spoilage microorganisms [2, 3, 5, 6]. Biofilms play a role in the deterioration of food, contributing to the degradation of food quality and sensory characteristics through the production of enzymes and metabolites. The protective matrix of biofilms poses a challenge for eradication, diminishing the effectiveness of traditional cleaning and sanitation methods [7, 8]. The resilience of biofilms can lead to recurrent contamination incidents, delays in production, and economic setbacks. Consequently, it is crucial to prioritize the development of innovative strategies and technologies aimed at preventing, controlling, and eliminating biofilms. This is essential for ensuring the safety and quality of food products throughout the entire production and storage process [7–9]. The review focuses on biofilm formation primarily in food processing environments, including biofilms growing on solid food products and equipment surfaces including storage tanks, reactors, and pipes. Biofilms on food surfaces pose a direct danger of contamination, while those on processing equipment can cause cross-contamination, making these systems crucial areas of concern [10].

In the processing, storage, and distribution of food, a number of methods have been proposed to prevent and control the formation of biofilms [9, 11, 12]. These studies demonstrate that there is not a single, all-encompassing way to combat the threat posed by biofilms. The factors influencing the catalytic functionality of biofilms exhibit variability based on the specific type of food, as well as biotic, abiotic, and edaphic elements, coupled with the conditions associated with food handling, processing, storage, and distribution, as indicated in the literature [6, 11–13]. Studies in the last decade [6, 8, 11, 12, 14] emphasize that surface activity emerges as a consistent factor underpinning the initiation, lifespan, and resilience of biofilms across diverse circumstances. Surface activity, denoting the ability of substances like surfactants to alter surface or interface characteristics, holds a crucial role in the biofilm realm. It significantly influences the initial attachment, growth, and maturation of microbial communities on surfaces [2, 3, 15, 16]. Microorganisms produce diverse surface-active compounds, including biosurfactants, exopolysaccharides, and lipids, facilitating interactions with surfaces and biofilm formation [3, 15]. These compounds enhance microbial adhesion by reducing surface tension, improving wetting, and fostering favorable interactions. Additionally, surface-active substances can modify biofilm surface properties, influencing structure, stability, and resistance to antimicrobial agents [3, 6, 15]. Understanding surface activity's role in biofilm formation provides valuable insights into microbial adhesion and biofilm development, aiding in the formulation of strategies for biofilm prevention or management across diverse environments.

Rheology, as a scientific discipline, is instrumental in comprehending biofilms' surface properties, structure, resistance to flow, and stability. Rheology, the study of how complex fluids respond under stress, is integral to understanding the behavior of substances like those in food processing. The study of material flow and deformation, rheology, significantly shapes biofilms' physical and mechanical properties. In the context of food, the physical domain, often referred to as the food matrix, is profoundly influenced by the flow and deformation of nanomaterials within it, consequently impacting the functionalities and behaviors of the food matrix. By examining rheological factors such as viscosity, elasticity, and yield stress, researchers can gain valuable insights into the adhesion, growth, and stability of biofilms on surfaces. This understanding of how rheology impacts biofilm development is pivotal in designing effective control strategies to prevent biofilm formation and developing targeted interventions for their eradication [17]. Understanding the influence of fluid flow, shear stress, and substrate properties on the initiation, structure, and functionality of biofilms has essential implications for food safety [18]. It helps develop targeted solutions to prevent their formation and improve medical treatments by creating better antimicrobial agents. Insights into their mechanical properties aid in designing interventions for industrial settings to minimize biofilm-related issues. Moreover, biofilm research sheds light on their vital role in environmental processes. Biofilm rheology significantly impacts industries, healthcare, and environmental management [19].

Moreover, the constant evolution of new strains of microorganisms adds another layer of complexity to the landscape. For example, International Space Station researchers recently discovered three new bacterial strains [20], showcasing the ongoing microbial diversity. Microorganisms, in their dynamic nature, also exhibit evolving resistance mechanisms, offering potential opportunities for the development of novel chemotherapeutic agents. Boudet et al. [21] underscore that the control of biofilm formation is intricately tied to specific strains, with varying influences, while studies by Nakanishi et al. [22] have addressed the interaction between biofilms and different surface materials, outlining how substrate properties influence biofilm adhesion and development. Wang et al. [23] and Kundukad et al. [24] have demonstrated the important effects of shear stress and fluid flow on biofilm formation; an in-depth comprehension of these variables under the conditions found in the food industry is still lacking. Specifically, non-Newtonian fluids, widespread in many food processes, are yet to be properly explored. The potential impact of rheology on biofilm formation, prevention, and control may differ based on the strain, exhibiting distinct characteristics and intensities [21]. However, despite the significance of these interactions, there is a notable scarcity of comprehensive reviews delving into the intricate interplay between rheology and biofilm formation. Specifically, the influence of fluid flow, shear stress, and substrate properties on the initiation, structure, and functionality of biofilms remains understudied.

This review is aimed at addressing this gap by adopting a novel approach that considers both past and recent research studies on the influence of rheology on biofilms. Such an approach is poised to unveil trends, identify research gaps, and propose avenues for further exploration in utilizing rheology to strategically mitigate biofilm-related risks in the food industry. Providing an extensive perspective, this review delves into the existing knowledge surrounding biofilm formation and the myriad factors contributing to their establishment and persistence. It explores the rheological characteristics of food matrices, the adhesion of pathogens to food surfaces, the nuanced influence of rheology on biofilm formation, the factors influencing the interplay between rheology and biofilms, the analytical methods employed to assess these interactions, and the broader implications for food safety across the supply chain. A comprehensive understanding of these aspects is imperative for the development of efficient and enduring strategies aimed at preventing the formation and persistence of biofilms in food processing and storage settings.

## 2. Rheology in the Food Industry

Rheology is the branch of science that studies the flow and deformation behavior of materials, particularly liquids and soft solids, under the influence of applied forces or stresses. It provides insights into the physical properties that determine how substances respond to forces, enabling us to understand and manipulate their behavior in various applications. Three fundamental parameters commonly used in rheological analysis are viscosity, elasticity, and yield stress. Viscosity refers to a material's resistance to flow and describes how easily a substance can be deformed under an applied force. It determines the thickness or consistency of a fluid and plays a significant role in processes such as mixing, pumping, and coating [25]. High-viscosity fluids, such as honey, are thick and resistant to flow, while low-viscosity fluids, such as water, flow easily. Viscosity changes can impact the density and thickness of biofilms, with lower viscosity environments frequently producing thicker and more resilient biofilm forms. For example, because there is more substrate available, biofilms in low-viscosity fluids might exhibit higher levels of metabolic activity [26]. Elasticity, on the other hand, measures a material's ability to return to its original shape after deformation. An elastic material can withstand stress and quickly return to its original form once the stress is removed [27]. This property is important in determining the texture and mouthfeel of many food products. For example, a gummy candy's elastic behavior contributes to its chewiness. Higher elasticity can help biofilms remain resilient by enabling them to keep their structural integrity in changing flow conditions. Higher elastic biofilms are found to be more stable in dynamic environments because they are unlikely to detach under shear stress [28].

Yield stress represents the minimum amount of stress required to initiate a flow transition in a material [29]. It is especially relevant in materials that exhibit a solid-like behavior until a certain threshold is exceeded, after which they flow like liquids. Yield stress is important in understanding the stability and “spreadability” of products such as spreads and emulsions, which need to maintain their structure under certain conditions but flow easily when used, such as salad dressing, ketchup, margarine, and mayonnaise. The technical explanation is that the internal nano- and microstructure resists the applied force and reversibly deforms [30]. The consequent structural breakdown in the process is different from both the original and the flow [30]. For biofilms, a higher yield stress implies that greater force is required to remove the biofilm from a surface, hence increasing adherence. According to Fabbri and Stoodley [31], biofilms with higher yield stress can remain attached to surfaces even under high fluid shear, making cleaning techniques more challenging.

Rheological measures quantify qualities such as viscosity and elasticity, which are critical for product quality in food sectors. Viscometers and rheometers measure fluid flow under different shear situations. For example, rotational viscometers measure yogurt viscosity to ensure a smooth texture [32]. Dynamic rheology, which employs oscillatory tests, analyzes both elastic and viscous components, which are critical for products such as dough or cheese [33]. Rheometers may also determine yield stress and elasticity in emulsions like mayonnaise [34]. In chocolate production, accurate rheology control affects mouthfeel, coating, and spreadability, hence boosting sensory experience and shelf life [35]. These rheological parameters are essential tools for characterizing and predicting the behavior of food matrices (the molecular relationships between the nutrient and nonnutrient components of food), aiding in product development, process optimization, and quality control across the food industry. The yielding phenomena and yield stresses distinguish the behavior of food materials into elastic deformation and viscous or viscoelastic flow [25]. Rheology is important for food safety because it helps to comprehend the physical properties that influence the development of biofilm, which can lead to contamination. By optimizing rheological characteristics, businesses in the food sector can more effectively manage biofilm-related risks, leading to safer food processing environments.

### 2.1. The Rheological Properties of Food Matrices

As noted above, the general definition of rheology includes the response of materials to applied force(s) or their flow characteristics at rest. These two perspectives are vital for the understanding of food rheology. In food processing, it is almost impossible not to apply force in the decomposition and recomposition involved in accessing/preparing food nutrients from plant or animal materials. Rheological characterization helps us understand the functional relation between deformation, stresses, and the resulting rheological properties such as viscosity, elasticity, and viscoelasticity [25].

Characterizing fluid behavior within biofilms has led to the development of various mathematical models to understand and describe their complex dynamics. These mathematical models simulate and predict fluids' behavior within biofilms, considering factors like flow, diffusion, and reaction kinetics occurring within these microbial communities. These mathematical models consider flow, diffusion, and reaction kinetics, which all occur within these microbial communities, to model and predict the behavior of fluids within biofilms. Hydrodynamic models, mainly concentrating on fluid flow inside the biofilm structure while considering various aspects such as fluid velocity profiles, shear stress, and pressure gradients within the biofilm matrix, are among the models connected to the fluid behavior of biofilms. Furthermore, fluid movement may impact waste clearance, nutrient delivery, and biofilm structure. Additionally, mass transport models explain how gases, nutrients, and signaling chemicals travel throughout the biofilm. They take into consideration the processes of diffusion or advection, examining how these materials travel through the biofilm matrix and affect the development and activity of microorganisms. In addition to biofilm structural models, which describe the physical structure of the biofilm and include the arrangement of microbial cells, extracellular polymeric substances (EPSs), and water channels, reaction kinetic models describe the biochemical reactions taking place within biofilms, such as metabolic processes, substrate utilization, and waste production. To simulate fluid behavior accurately, they could use knowledge of biofilm structure [36]. A recent investigation has presented a mathematical framework delineating the motility patterns of unbound cells within biofilms formed via dispersion mechanisms. These mechanisms serve as a survival strategy for microbial species, facilitating biofilm relocation onto new surfaces amidst adverse environmental conditions. However, in certain scenarios, biofilm dispersion contributes to the disseminating pathogenic bacteria within human systems. Notably, D'Acunto et al. [36] offer a theoretical model elucidating the interactive response triggered by environmental cues. Continuous models in their research scrutinize the intricate interplay between environmental stimuli instigating cell detachment, encompassing factors such as nutrient scarcity, exposure to toxins, and endogenous biocidal agents. This model comprehensively assesses and prognosticates biomass reduction resulting from two inherently distinct modes—biofilm dispersion involving cellular detachment and the mechanical separation influenced by shear forces. Furthermore, this model intricately tracks the dynamics of sessile microbial species within the biofilm matrix and the mobility of dispersed free cells released into the surrounding liquid phase following biomass inactivation [36].

The study of rheological properties in food matrices involves investigating the flow and deformation behavior of complex materials such as gels, emulsions, and suspensions. These properties play a crucial role in determining the texture, stability, and sensory perception of various food products. Researchers are able to comprehend the structural interactions of ingredients, processing methods, and environmental conditions by analyzing factors like viscosity, elasticity, and yield stress, thus facilitating the formulation of optimized food products with desired sensory and functional attributes. This multidisciplinary field combines principles from physics, engineering, and food science to enhance our understanding of how the physical properties of food matrices influence their processing, quality, and consumer acceptance.

### 2.2. Food Matrices and Their Rheological Properties

In the food sector, texture and mouthfeel are essential factors that influence consumer choices and the quality of the product. The rheological characteristics of biofilms are crucial in determining these sensory features because they influence how food items interact with sensory receptors while eating. Furthermore, processing and manufacturing conditions are usually designed to maximize these characteristics, demanding a full understanding of how biofilm behavior affects these processes. This section will look at the complex interaction between biofilm rheology and sensory characteristics, emphasizing the consequences for food composition, processing techniques, and ultimate product appeal. The diversity of food matrices is vast, ranging from liquid beverages to semisolid gels, emulsions, suspensions, and solid structures. Each food matrix possesses unique rheological properties that arise from the interactions between its components, such as proteins, fats, carbohydrates, and water. Understanding the rheological behavior of these diverse matrices has received research attention [8, 12, 25, 27, 29].

#### 2.2.1. Texture and Mouthfeel

Rheological properties directly influence the texture and mouthfeel of food products. For instance, the creamy texture of ice cream results from a delicate balance between the viscosity of the ice cream base and the formation of small ice crystals. In bread making, dough rheology affects the final crumb structure and overall sensory experience.

#### 2.2.2. Processing and Manufacturing

The rheological properties of food matrices influence processing operations. The flow behavior of liquid food products affects filling, pumping, and mixing operations. Non-Newtonian fluids, like ketchup or mayonnaise, have viscosity that changes with shear rate, making their processing and dispensing challenging.

Several investigations utilizing different rheological and textural approaches have been conducted to analyze the fundamental structure of mozzarella, pizza cheese, and similar food products [37–40]. The investigation conducted by the authors in the food industry, particularly in the context of mozzarella cheese processing, highlighted a significant reliance on total shear work input concerning rheological properties and melt functionality. Their findings revealed a distinct nonlinear trend characterized by an escalation in the consistency coefficient and apparent viscosity, coupled with a concurrent decrease in the flow behavior index. This observed trend correlated with the augmentation of accumulated shear work, indicative of a phenomenon termed as work thickening behavior [41].

A comprehensive examination using diverse rheological methods was conducted on pizza cheese, which served as an exemplary anisotropic substance. Dahl et al.'s study [39] involved the assessment of pizza cheese at distinct processing stages—premixing, during mixing, poststretching, and after 1–2 weeks of storage. The synergy between rheological and microstructural analyses was highlighted in their research, revealing nuanced network variations among samples across diverse processing steps. This approach contributes to the enhancement of the understanding of anisotropy across varying length scales [39].

#### 2.2.3. Stability and Shelf Life

Understanding the rheology of emulsions and suspensions is crucial for product stability and shelf life. Many food products, like salad dressings and dairy beverages, are colloidal systems where the separation of phases can be prevented or controlled by manipulating their rheological properties.

#### 2.2.4. Sensory Perception

Rheological properties significantly impact the sensory perception of food. The “mouth-coating” sensation of a creamy soup, the “crispiness” of a potato chip, or the “slurpability” of a noodle soup is influenced by the rheological behavior of the food matrix.

#### 2.2.5. Nutrient Delivery

The rheology of food matrices can affect the release and absorption of nutrients during digestion. For example, the thickness of a liquid may impact how quickly it leaves the stomach and enters the intestines, affecting nutrient absorption rates.

#### 2.2.6. Innovation and Product Design

Manipulating rheological properties allows for innovation in food product design. Creating novel textures, such as foams, aerated gels, or structured emulsions, enhances the culinary experience and opens up new avenues for product development.

#### 2.2.7. Health and Wellness

Rheology can impact the perceived healthiness of food. For instance, the mouth-coating sensation in low-fat products can be enhanced using hydrocolloids to mimic the sensation of higher fat content, potentially influencing consumer preferences.

The diverse rheological properties of food matrices play a pivotal role in determining the sensory, processing, and stability characteristics of food products. By understanding and tailoring these properties, food scientists and engineers can create products with improved quality, functionality, and consumer acceptance.

### 2.3. Influence of Composition, Processing Conditions, and Temperature on Rheological Properties of Food

Rheological properties are complex and multifaceted, influenced by a combination of various factors; some of which are general, while others are circumstantial. This discussion is limited to the general influence of composition, processing conditions, and temperature, on the rheological properties of food which is reviewed.

#### 2.3.1. Composition

##### 2.3.1.1. Particle Size and Distribution

The size and distribution of particles in a material can significantly impact its rheological properties [42]. Smaller particles can lead to increased viscosity and resistance to flow due to their tendency to form stronger networks. Furthermore, consistent particle size can allow for a smoother flow, which could encourage the retention of nutrients necessary for the establishment of biofilms [43].

##### 2.3.1.2. Particle Concentration

Higher concentrations of particles, such as fillers, additives, or thickeners, can lead to more pronounced interactions between particles, resulting in increased viscosity and shear-thinning behavior [44, 45]. Higher particle concentrations can promote the agglomeration of microorganisms and biofilm formation by increasing interactions between microorganisms and particles [46]. This can result in a more stable biofilm structure.

##### 2.3.1.3. Polymer Molecular Weight

In polymer solutions, higher molecular weight polymers tend to exhibit higher viscosity due to increased entanglement and chain interactions [47]. Higher concentrations of particles can create a more favorable environment for microbial aggregation and biofilm formation due to increased interactions among microorganisms and particles [48].

##### 2.3.1.4. Solvent or Liquid Content

The type and amount of solvent or liquid in a mixture can affect the viscosity and flow behavior. Higher solvent content can reduce viscosity, while lower solvent content can lead to thicker materials [49]. Water activity in food products affects bacteria growth and biofilm formation. High solvent content can dilute nutrients, while low water content can enhance nutrient concentration, influencing biofilm dynamics [15].

#### 2.3.2. Processing Conditions

##### 2.3.2.1. Shear Rate

The rate at which shear forces are applied during processing can alter the rheological behavior. Materials might exhibit shear-thinning (viscosity decreases with increasing shear rate) or shear-thickening (viscosity increases with increasing shear rate) behavior [50]. Processing-related shear pressures have the potential to either break up already-existing biofilms or encourage the development of new ones by promoting microbial attachment to surfaces [51]. Under various shear situations, biofilms can display distinct growth patterns that impact their structural integrity and resistance to removal.

##### 2.3.2.2. Mixing Time and Intensity

Mixing intensity and time can impact the degree of particle dispersion, leading to changes in viscosity and other rheological properties, as increased intensity and longer mixing durations often enhance particle distribution and reduce agglomeration, resulting in a more homogeneous mixture [52]. Increased mixing intensity and time can improve particle dispersion, resulting in a more stable environment for the growth of microorganisms and biofilm formation [53]. Proper distribution of nutrients and microbes is critical for optimal development of biofilm.

##### 2.3.2.3. Deformation History

Materials subjected to different processing histories may show varying rheological behaviors. Thixotropic materials, for example, regain viscosity over time after being subjected to shear, impacting the stability of biofilms formed on such surfaces [54–56].

##### 2.3.2.4. Curing and Crosslinking

In some systems, curing or crosslinking reactions can alter the molecular structure, resulting in changes in viscosity, elasticity, and flow behavior [57]. These processes can produce a matrix that either inhibits or promotes microbial attachment, influencing biofilm formation and stability [58].

#### 2.3.3. Temperature

##### 2.3.3.1. Viscosity–Temperature Relationship

Changes in temperature can significantly affect the viscosity of materials. Most fluids exhibit decreased viscosity with increasing temperature, although this trend might not hold true for all materials. Temperature fluctuations can influence the viscosity of food matrices, influencing how easily microbes disperse and form biofilms. Increased temperatures usually reduce viscosity, potentially increasing the movement and attachment of microorganisms [59].

##### 2.3.3.2. Phase Transitions

Temperature changes can cause phase transitions, such as melting or crystallization, which can alter the internal structure of materials and impact their rheological properties. Melting facilitates biofilm formation by increasing surface roughness and moisture [60].

##### 2.3.3.3. Polymer Behavior

Polymers can exhibit different rheological behaviors at different temperatures due to changes in polymer chain flexibility, entanglement, and interactions. Changes in the flexibility and entanglement of polymer can encourage microbial adherence by making the surface softer and more accessible or discourage microbial adherence by establishing a hard, tightly packed matrix that inhibits attachment and nutrient diffusion [61, 62].

## 3. Pathogen Adhesion to Food Surfaces

Foodborne pathogens pose a persistent threat to food safety, and understanding the intricacies of their adhesion to various surfaces is crucial for effective control measures. In this section, we delve into the diverse mechanisms that govern pathogen adhesion to different food surfaces. We explore the impact of hydrodynamic forces and surface roughness on adhesion dynamics and, importantly, dissect the role of rheology, considering the effects of flow characteristics and viscoelasticity.

### 3.1. Mechanisms of Pathogen Adhesion to Different Food Surfaces

**Figure 1:**
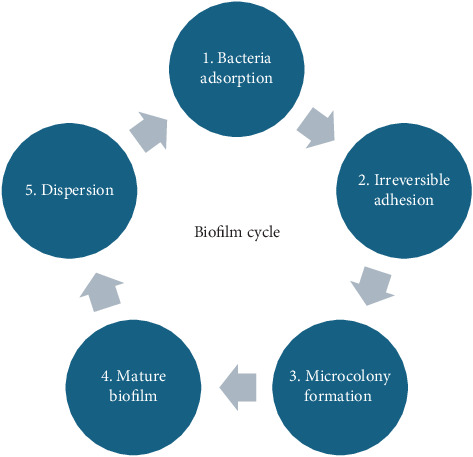
Biofilm cycle.

The adhesion of pathogens to food surfaces is a multifaceted process dictated by intricate molecular interactions [63]. Understanding the molecular interactions involved in pathogen adhesion and biofilm formation provides a foundation for developing targeted preventive strategies. Foodborne pathogen adherence to food surfaces poses major food safety risks, including contamination and foodborne disease. The biofilm formation cycle encompassing bacteria adsorption, irreversible adhesion, microcolony formation, mature biofilm development, and eventual dispersion is critical in understanding how pathogens persist on surface (Figure 1). For example, Oliveira et al. [64] reported that *Staphylococcus aureus* can form biofilms on catheter surfaces, resulting in recurrent infections. Similarly, *Salmonella* biofilms have been shown to develop on food contact surfaces, such as cutting boards and stainless steel [65], demonstrating the protective habitat biofilms provide for pathogens. Biofilms create a protective environment for pathogens, increasing their resistance to sanitization treatments and resulting in higher survival rates on food contact surfaces. The formation of biofilms can also allow the transmission of genetic material between bacteria, potentially leading to the creation of more aggressive strains or antibiotic resistance. Biofilms of antibiotic-resistant *Enterococcus faecalis* could transfer resistance genes to other bacteria in the presence of biofilms, contributing to the establishment of multidrug-resistant strains [66].

Surface characteristics play a crucial role in facilitating adhesion, with hydrophobic interactions constituting a prominent factor in the initial approach of pathogens to surfaces [67]. The electrostatic charge of both the pathogen and the food surface further contributes to specific interactions, culminating in irreversible attachment [63]. Environmental factors, including ionic strength [68] and surface roughness [69], significantly influence the adhesion process. While there is a broad understanding of common adhesion mechanisms, the diversity in pathogen strains and food surfaces demands a nuanced approach. Some pathogens might rely on specific ligand–receptor interactions, while others could be more influenced by surface charge or hydrophobicity [70].

Pathogens such as *Listeria monocytogenes* and *Salmonella enterica* exhibit strain-dependent variations in their adhesion mechanisms [71]. Silva et al. [72] and Oliveira et al. [73] demonstrated that the adhesion of these pathogens to materials commonly found in kitchens is influenced by the specific strain of the pathogen and the composition of the food surface. Surface appendages such as flagella, fimbriae, and pili play a vital role in anchoring pathogens to surfaces [74]. Moreover, spores, characterized by their inherent hydrophobicity and surface coverage with hair-like structures, exhibit an enhanced rate of adhesion [75].

The composition and microstructure of surfaces, including stainless steel, glass, and polymers, significantly influence adhesion outcomes. Glass surfaces, commonly employed in food processing, are recognized for their hydrophilic nature, which may deter pathogen adhesion [76]. Conversely, stainless steel surfaces, prevalent in food equipment, may exhibit regions of hydrophobicity, necessitating surface treatment to mitigate the risk of colonization by pathogens [77]. Polymer-based surfaces, contingent on their hydrophobic or hydrophilic attributes, distinctly influence pathogen adhesion [78], underscoring the importance of surface modifications to enhance resistance. The interaction between bacterial surface structures and the physicochemical properties of the materials is complex and varies across different food surfaces [79].

### 3.2. Role of Hydrodynamic Forces in Bacteria Adhesion

The adhesion of bacteria to surfaces is a complex process influenced by various factors, including hydrodynamic forces. Fluid dynamics, characterized by the flow of liquids or gases, plays a crucial role in shaping bacterial behavior on surfaces, affecting biofilm architecture, composition, and mechanical strength [80, 81].

Under dynamic conditions, biofilm growth is facilitated, as observed in various environments such as the oral cavity and catheter microenvironments [63]. The continuous flow of saliva and gingival crevicular fluid in the oral cavity exposes dental plaques to dynamic conditions, influencing their development, biofilm formation, structure, bacterial metabolism, and stability on tooth surfaces through changes in nutrient availability and shear forces (the physical movement or force applied to the biofilm by the flowing fluid) [82]. Biofilm growth is facilitated under dynamic conditions because the constant flow of liquids provides shear forces that promote bacterial adhesion and biofilm formation [83]. Dynamic conditions are common in industrial settings, including piping systems, reactors, and other equipment with continuous or intermittent fluid flow [84]. Heat exchangers and membrane bioreactors are examples of dynamic processing units in which biofilms thrive due to the shear forces produced by fluid movement [85]. In contrast, static circumstances occur in storage tanks, vats, or other stationary vessels where there is little to no fluid movement, resulting in a more quiescent surface [86]. These static settings have little shear stress but can nevertheless sustain biofilm formation, depending on the availability of nutrients and surface characteristics.

In catheter microenvironments, fluid flow significantly influences biofilm formation [80]. Studies have demonstrated that the shear stress induced by the laminar flow of finite-sized fluid particles in the surrounding microenvironment enhances biofilm formation by increasing EPS production and strengthening the EPS matrix in bacteria like *Staphylococcus aureus* [87]. The resulting EPS matrix serves a protective role, allowing biofilms to recover from mechanical challenges induced by pressure and flow, creating more resistant and compressible biofilms [88].

Biofilms under shear stress from the physical movement or force by a flowing fluid exhibit increased expression of molecules involved in signal transduction, improved oxygenation, and enhanced bacterial growth. The positive feedback loop created by fluid shear initiates biofilm formation by stimulating EPS production and providing nutrients for growth [89]. Studies using a parallel plate flow chamber have shown that multispecies biofilms, subjected to dynamic flow, become more resistant to compressive forces compared to single-species biofilms [90]. The dynamic conditions result in biofilms that are more elastic, resistant, denser in matrix proteins, and EPS [91].

The human mouth serves as a relevant model for studying the influence of dynamic flow conditions on bacterial adhesion and biofilm formation due to continuous salivary flow. Studies utilizing artificial mouth models with continuous saliva flow have revealed that biofilm viability is significantly higher under semidynamic conditions compared to static conditions [92].

Despite the observed positive impact of dynamic flow on biofilm growth, contradictory findings have been reported. Some studies show a lower degree of biofilm formation under dynamic conditions [93]. The disparity in results may be attributed to variations in bacterial species, flow types, shear stress magnitudes, and surface properties. While dynamic conditions encourage biofilm formation, there are scenarios where static conditions lead to better biofilm formation. This can be linked to reduced mechanical disruption and constant accumulation of nutrient on the surface, which creates an ideal environment for bacterial colonies [94]. Bacteria are not subjected to flow or shear forces in static conditions, which could lead them to be detached from surfaces. Furthermore, biofilms in static conditions can generate more extensive three-dimensional structures as a result of unrestricted growth, which promotes their persistence [95].

Hydrodynamic conditions can significantly affect the antiadhesion properties of surfaces. Superhydrophobic surfaces, characterized by increased water contact angle and roughness, exhibit enhanced antiadhesion properties under flow conditions [96]. The entrapment of air in the hydrophobic layer minimizes bacterial contact with the surface, resulting in improved reduction of bacterial adhesion.

### 3.3. Impact of Rheology on Pathogen Adhesion: Flow Characteristics and Viscoelastic Effects

Rheological properties play a pivotal role in governing the adhesion dynamics of microbial pathogens, with flow characteristics and viscoelasticity emerging as critical factors in shaping these interactions [97, 98]. The adherence of pathogens, particularly *Pseudomonas aeruginosa* strains, is intricately linked to the rheological attributes of their EPS [99].

Flow characteristics such as laminar versus turbulent flow and the magnitude of shear stress significantly influence pathogen adhesion. The constant flow of fluid in laminar flow encourages the uniform distribution of nutrients and bacterial cells, which makes adhesion easier in low-shear areas [100]. On the other hand, because of the chaotic motion of fluid particles, turbulent flow can prevent adhesion [101]. This results in significant shear stress, which physically removes pathogens from the surface. The flow regime profoundly impacts pathogen adhesion, as elucidated by quartz crystal microbalance with dissipation (QCM-D) experiments conducted in various ionic strength conditions [97]. The calculated Péclet number, indicative of a diffusion-dominated flow regime, suggests that the adhesion of EPS to the QCM-D sensor is diffusion limited. Notably, an elevation in ionic strength led to increased adhesion, aligning with Derjaguin–Landau–Verwey–Overbeek (DLVO) theory principles which describes the interactions between van der Waals forces, which are attractive, and electrostatic repulsion. According to Orgad et al. [97], the screening of the electrical double layer reduces repulsive forces, facilitating stronger interactions between the negatively charged silica surface and EPS components. Moreover, the flow-induced changes in fluid viscosity are reflected in the measured frequency shifts during injection stages, indicating a diffusion-limited adherence mechanism.

Biofilms' viscoelastic qualities such as their capacity to deform and recover under stress have an impact on pathogen adherence. Biofilms with strong viscoelastic properties can survive shear stresses caused by fluid movement, making them more resilient in dynamic situations. Pathogens such as *Pseudomonas aeruginosa* develop EPS with high viscoelasticity [98], which allows the matrix of the biofilm to absorb and dissipate mechanical stresses. This improves the ability of biofilms to withstand in flow conditions and to adhere despite mechanical forces. Inhibiting pathogen adhesion will need high-shear, turbulent flow regimes that disrupt early attachment of bacteria [101]. Furthermore, altering the viscoelastic properties of biofilms, such as altering the production of EPS or degrading matrix components, can diminish the biofilm's mechanical resistance, making it more sensitive to shear pressures and easily detached [102].

## 4. Rheological Impact on Biofilm Formation

### 4.1. Initial Attachment and Colonization of Pathogens on Food Surfaces

The rheological properties of the environment play a crucial role in the initial attachment and colonization of pathogens on food surfaces. Understanding the interplay between rheology and biofilm formation is vital for devising effective strategies to mitigate foodborne pathogen contamination. Numerous studies have investigated how factors such as surface viscosity and adhesion forces influence the initial stages of biofilm formation [103, 104]. The biofilm formation process is in a cycle of five stages (Figure 1): initial reversible attachment, where the first contact occurs between the surface and the moving planktonic bacteria; irreversible attachment, where the bacteria anchor themselves more permanently; maturation, where the biofilm establishes and may change in size and shape; microcolony formation, where bacteria grow and form microcolonies; and dispersion, where the biofilm disperses [105]. The rheological characteristics of surfaces can impact the transport and deposition of bacterial cells, influencing the adherence process [106].

Ruengvisesh et al. [107] demonstrated that surfaces with cracks, pockets, crevices, and native openings promote enhanced initial attachment of pathogens. This underscores the importance of considering not only the chemical composition but also the mechanical aspects of food matrices in preventing microbial contamination.

### 4.2. Biofilm Development and Maturation in Relation to Rheological Properties

As biofilms progress from initial attachment to maturation, the rheological properties of the microenvironment continue to exert a profound influence on their development. The viscoelastic nature of the matrix affects bacterial motility, nutrient diffusion, and the overall metabolic activity within the biofilm community [108]. Studies have shown that biofilm growth is not solely dictated by biochemical cues but is significantly modulated by the rheological characteristics of the surrounding medium [109].

Cao et al. [110] elucidated the dynamic interplay between rheology and biofilm maturation. The researchers observed distinct phases in biofilm development corresponding to changes in the rheological properties of the environment. The study noted that as biofilms develop, distinct phases that coincide with changes in the rheological properties of the environment take place. This shows that the physical characteristics of the environment significantly impact on the function and structure of biofilms. As biofilms mature, their rheological properties, including viscosity and elasticity, also change. This could be attributed to the secretion of EPS by biofilms as they grow; the presence of EPS affects the viscosity and elasticity of the surrounding matrix [111]. These changes influence various aspects of biofilm behavior, including attachment, dispersal, and resistance to antimicrobial agents. For example, increased viscosity may make it more difficult for antibiotics to penetrate the biofilm matrix, leading to greater resistance to treatment [112]. Understanding these rheological dynamics is imperative for predicting biofilm growth trajectories and devising interventions to disrupt the maturation process.

### 4.3. Impact of Rheology on Biofilm Architecture

Rheological properties profoundly impact the architecture of mature biofilms, influencing key structural parameters such as thickness [104]. The viscoelasticity of the microenvironment determines the extent of biofilm expansion and vertical growth. Studies by Saint Martin et al. [113] and Krsmanovic et al. [104] demonstrated that changes in the consistency or flow velocities directly correlate with variations in biofilm thickness, emphasizing the role of rheology as a critical regulator of biofilm architecture.  Table 1 presents an overview of rheological modifications that can be exploited to limit or control the initiation and/or functionality of biofilms. Literature(s) that have spotlighted each overview is/are indicated in the table.

It is important to note that the specific impact of these modifications on biofilms can vary depending on the type of microorganisms involved, the surface characteristics, and the overall environmental conditions. Findings reported in studies reviewed are based on the desired outcomes for a particular application, such as preventing biofilm formation on medical devices or controlling biofouling in industrial settings.

The microstructure of biofilms, encompassing the spatial arrangement of bacterial cells and EPS, is intricately tied to rheological properties [120]. Advances in imaging techniques have enabled researchers to correlate rheological alterations with changes in biofilm microstructure. Charlton [121] employed advanced microscopy to unveil the intricate relationship between rheology and the spatial organization of biofilm components.

## 5. Factors Modulating Rheology–Biofilm Interactions

### 5.1. Role of EPSs in Biofilm Formation and Their Relation to Rheology

Biofilm formation is a complex process influenced significantly by the composition and characteristics of EPS [122]. EPSs play a crucial role in the stability and structure of biofilms, impacting their rheological properties [115]. Several studies have explored the correlation between EPS composition and biofilm rheology [121, 123].

Research by Seviour et al. [124] identified biofilms as hydrogels through rheological studies, establishing a connection between EPS and the mechanical properties of biofilms. The degradation of specific EPS components, such as proteins and *α*-polysaccharides, resulted in a loss of storage modulus, indicating their substantial role in maintaining biofilm stability. This finding contrasts with previous claims that *β*-polysaccharides play a primary role, emphasizing the need for a comprehensive understanding of the specific EPS molecules influencing biofilm rheology.

The rheological properties of biofilms, as influenced by EPS, have significant implications for biofilm-based applications, including wastewater treatment and environmental bioremediation [125]. By elucidating the mechanical aspects of EPS-rich biofilms, researchers can devise strategies to enhance biofilm stability and performance.

### 5.2. Effect of Nutrients and Growth Conditions on Biofilm Rheology

Nutrient availability and growth conditions significantly impact the rheological characteristics of biofilms. Growth conditions including temperature, pH, nutrient availability, and oxygen levels all play important roles in biofilm development. Elevated temperatures within a species' optimum range tend to speed up microbial metabolism and EPS synthesis, encouraging biofilm formation [126]. Similarly, pH influences biofilm stability and microbial activity, with acidic conditions favoring staphylococcal biofilm formation [127]. Nutrient-rich conditions promote denser biofilm matrix, but nutrient deficiency frequently results in thinner, less stable biofilms [128]. Fluid flow creates shear stress, which promotes biofilm formation by aiding or preventing cell attachment [89].

The composition and distribution of EPS, which form the structural matrix of biofilms, vary in response to environmental factors. Studies by Zhang et al. [129] observed a higher percentage of extracellular proteins related to catalytic activity in flocs compared to granules. This difference in protein content influenced the degradation of the EPS matrix, leading to variations in biofilm structure and, consequently, rheological properties.

Additionally, the work of Martín-Cereceda et al. [130] highlighted the impact of growth conditions on the concentration of extracellular proteins in biofilms. Understanding the impact of nutrient fluctuations on EPS composition is crucial for predicting and controlling biofilm rheology. Insights into how growth conditions shape the structural elements of biofilms provide a foundation for optimizing biofilm performance in diverse settings. This knowledge aids in tailoring biofilm-based processes for specific applications and environmental conditions.

### 5.3. Impact of Shear Forces and Flow Patterns on Biofilm Stability and Detachment

Shear forces and fluid flow patterns play a pivotal role in determining biofilm stability and detachment [131]. Flow patterns, which can be laminar or turbulent, explain how fluids travel in a given system. Laminar flow is smooth and ordered, whereas turbulent flow is chaotic, resulting in significant mixing. Fluid type impacts on the stability of biofilms because Newtonian and non-Newtonian fluids have different flow characteristics and interact with biofilm structures differently. Newtonian fluids, which have a constant viscosity regardless of applied stress, produce consistent shear forces throughout biofilms, impacting the process of detachment in a predictable manner [132]. Conversely, non-Newtonian fluids can produce localized stress variations within biofilm structures, particularly those that exhibit shear-thinning characteristics (where viscosity decreases with increasing shear rate) [133, 134]. This can either improve biofilm adhesion under low shear conditions or encourage detachment as shear rates increase, because the lower viscosity allows for more fluid flow and transmission of stress through the biofilm matrix. The interaction between biofilms and the surrounding fluid is influenced by the rheological properties of EPS [135]. These patterns are especially important in understanding biofilm behavior in systems such as piping, which frequently experience laminar and turbulent flow depending on their flow rate and fluid type. Tanks, on the other hand, may display laminar flow depending on their mixing and operational conditions. These flow pattern differences have a direct impact on how biofilms develop, grow, and detach; hence, understanding fluid dynamics is critical for biofilm management in industrial settings.

**Figure 2:**
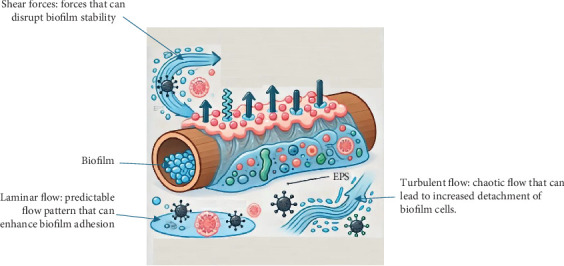
Influence of EPS on biofilm behavior and response to shear forces and flow patterns.

The molecular structure of EPS, specifically *β*-polysaccharides, plays a crucial role in biofilm stability under various flow conditions.  Figure 2 is used to depict the importance of the relationship between EPS and rheology; the EPS matrix modulates biofilm behavior by affecting its viscoelastic properties and its response to shear forces and flow patterns. Chen et al. [136] conducted a quadruple staining on EPS components, revealing the distribution of *β*-polysaccharides throughout the interior of granules. The rigid molecular structure of *β*-polysaccharides suggests their potential role in resisting shear forces and maintaining biofilm stability. On the other hand, Caudan et al. [137] demonstrated that enzymatic hydrolysis of specific polysaccharides affected the stability of aerobic granules. This underscores the importance of rheological studies in predicting how biofilms respond to mechanical forces. Understanding the interplay between shear forces, flow patterns, and biofilm rheology is essential for developing strategies to mitigate biofilm detachment and enhance the stability of biofilm-based systems.

## 6. Analytical Techniques for Assessing Rheology–Biofilm Interactions

Understanding the intricate interplay between rheology and biofilm formation necessitates a comprehensive analytical approach, integrating diverse experimental methods such as rheometry, microscopy, and molecular biology techniques. This section provides a scholarly examination of these methods, elucidating their underlying principles, merits, and constraints.  Table 2 summarizes the analytical techniques for assessing rheology–biofilm interactions as discussed in this section.

### 6.1. Overview of Experimental Methods

#### 6.1.1. Rheometry

Rheometry, as a central pillar in the analytical framework, plays a crucial role in unraveling the dynamics of rheology–biofilm interactions [138]. Rheometry involves the quantification of the mechanical properties of materials under various conditions. Specifically, in the context of biofilm research, interfacial rheological time sweeps offer a dynamic perspective on the evolution of biofilm strength over time. In the study conducted by Rühs et al. [139], this method proved instrumental in characterizing the transient elastic behavior of *Pseudomonas putida* KT2442, at the water–oil interface. The resulting data revealed distinct differences in biofilm formation kinetics and strength among the bacterial strains.

Amplitude sweeps, another facet of rheometry, provide structural insights by examining shear thickening or thinning behavior within biofilm layers [140]. The Lissajous plots generated through amplitude sweeps offer a rheological fingerprint for each adsorption layer [139]. Rühs et al. [139] used amplitude sweeps to quantify structural differences between the protein network formed by Luria–Bertani (LB) medium, the bacterial cell adsorption layer of *P. putida* KT2442, and the biofilm of *P. putida* KT2442. This approach allowed for the differentiation of viscoelastic networks and the identification of shear thickening or thinning behavior within these layers.

Rheometry emerges as a potent tool for quantifying the mechanical intricacies of biofilms, offering real-time monitoring capabilities. Liou et al. [141] emphasize the utility of rheometry in capturing elastic moduli and shear thickening behavior during biofilm formation. Real-time monitoring allows researchers to observe the dynamic evolution of biofilm properties, providing insights into the kinetics of adhesion and network formation.

However, rheometry has its limitations. The method predominantly focuses on physical properties, such as elasticity and viscosity, providing a macroscopic view of biofilm behavior [142]. To obtain a more comprehensive understanding, researchers often need to integrate rheometry with other techniques, such as microscopy and molecular biology approaches. Additionally, the interpretation of rheological data requires careful consideration of the environmental conditions, as changes in pH and ionic strength can influence the rheological properties of biofilms.

#### 6.1.2. Microscopy

Microscopy techniques, such as confocal laser scanning microscopy (CLSM) and light microscopy, complement rheometry by providing visual insights into the molecular architecture of biofilms. CLSM, with its high-resolution imaging capabilities, enables researchers to observe biofilm growth in real time at the water–oil interface [143]. CLSM was utilized to characterize bacterial strains and demonstrate their growth at the water–oil interface [139]. The resulting images highlighted the differences in biofilm formation among *P. putida* KT2442, *Salmonella typhimurium*, and *Escherichia coli*, with *P. putida* KT2442 forming a thicker and more even biofilm compared to the other strains.

Light microscopy, on the other hand, offers morphological assessments, aiding in the characterization of biofilm structures [144]. The combination of CLSM and light microscopy has been used for a comprehensive visualization of biofilm formation, confirming and complementing the rheological data [139]. This integrative approach enhances the interpretative capacity of the study, providing both quantitative and qualitative information on biofilm dynamics.

Microscopy techniques, while providing qualitative insights into biofilm morphology, encounter limitations in terms of two-dimensional observations and challenges in real-time visualization [145]. Harrison et al. [146] acknowledge these constraints, emphasizing the method's reliance on staining and labeling, which may introduce artifacts. The inherent limitations of light microscopy in providing detailed three-dimensional information necessitate complementary techniques for a more holistic characterization of biofilm structures.

Despite these limitations, the visual data obtained through microscopy augments the broader analytical framework. CLSM, in particular, offers the advantage of high-resolution imaging, allowing researchers to observe the spatial distribution of bacteria within biofilms [147]. The integration of microscopy with rheometry provides a multidimensional view of biofilm dynamics, combining the strengths of both techniques for a more nuanced understanding.

#### 6.1.3. Molecular Biology Approaches

Molecular biology techniques, particularly electrophoretic mobility measurements, contribute molecular-level information to the analytical framework. Electrophoretic mobility is a measure of the mobility of charged particles in an electric field, and in the context of biofilm research, it serves as a valuable tool for assessing bacterial interactions at the oil phase [148]. Javan Roshtkhari [149] highlighted the utility of electrophoretic mobility in gauging surface charge and hydrophobicity dynamics, offering insights into the potential of bacteria to adsorb at the oil phase.

The molecular-level understanding provided by electrophoretic mobility measurements complements the macroscopic data obtained through rheometry and microscopy [150]. This approach bridges the gap between the mechanical properties of biofilms and the underlying molecular interactions. For example, Rühs et al. [139] used electrophoretic mobility measurements to assess the hydrophobicity of different bacterial strains against mineral and medium-chain triglyceride (MCT) oil. The resulting data demonstrated a correlation between measured hydrophobicity, rheological properties, and microscopy images, showcasing the integrated nature of the analytical approach.

Molecular biology techniques, particularly electrophoretic mobility measurements, furnish invaluable molecular-level insights into bacterial interactions. Rühs et al. [139] underscore the method's efficacy in elucidating surface charge and hydrophobicity dynamics, providing a molecular basis for bacterial adhesion to the oil phase. The ability to assess hydrophobicity against different oil phases enhances the specificity of the analysis, allowing researchers to discern subtle variations in bacterial behavior.

However, molecular biology approaches fall short in providing direct insights into the mechanical properties of biofilms. Electrophoretic mobility, while informative about surface interactions, offers a limited perspective on the overall rheological behavior of biofilms [151]. To address this limitation, an integrative approach that combines molecular biology techniques with rheometry and microscopy becomes imperative. Additionally, the reproducibility of electrophoretic mobility measurements requires meticulous control of experimental conditions, and variations in these conditions can influence the reliability of the results.

In synthesis, the amalgamation of rheometry, microscopy, and molecular biology approaches enriches the depth and breadth of investigations into rheology–biofilm interactions. The triad of methods, each contributing unique perspectives, ensures a nuanced exploration of the physical, structural, and molecular facets of biofilm formation.

#### 6.1.4. Metabolic Assays and Metabolite Profiling

Metabolic activity is an important factor of microbial biofilm formation and resilience, regulating not just the rate of growth but also the composition of EPS that make up the biofilm matrix [152]. The MTT (3-[4,5-dimethylthiazol-2-yl]-2,5 diphenyl tetrazolium bromide) assay, resazurin assay, and ATP bioluminescence have all been used to examine microbial metabolism in biofilm research [153, 154]. These assays assess cellular respiration or the production of energy, providing information about the overall metabolic condition of biofilm-forming microorganisms.

Metabolite profiling techniques, including gas chromatography–mass spectrometry (GC-MS) and liquid chromatography–mass spectrometry (LC-MS), can detect the metabolites secreted by biofilms, which are essential for understanding their resilience and functionality [155]. For example, metabolite profiling has been used in identifying compounds involved in quorum sensing, a mechanism by which microorganisms control biofilm development based on population density [156]. By using these techniques, researchers can establish a link between microbial communities' metabolic activity and biofilm mechanical properties such as elasticity and viscosity, as well as how they respond to external stresses such as shear forces [108].

Metabolic assays have the advantage of offering direct information about the active state of microbial populations within biofilms. For example, ATP bioluminescence tests can provide real-time measurements of microbial activity, allowing for the early detection of biofilm formation [157]. These assays are especially relevant in industrial settings where quick biofilm detection is required for prompt response. However, metabolite profiling can be complex, requiring special equipment such as mass spectrometers, which may not be readily available in all study settings. Furthermore, the relationship between metabolic activity and biofilm development is not always clear, as biofilm structure varies according to environmental circumstances and microbial species [158]. To gain a thorough understanding of biofilm dynamics, a comprehensive approach combining metabolic assays with rheological, microscopic, and molecular biology approaches is required.

### 6.2. Integrating Analytical Techniques for Comprehensive Biofilm Monitoring and Management in Industrial Settings

To completely comprehend biofilm mechanics, these analytical techniques can be combined. We have seen that rheometry exposes macroscopic features like elasticity and viscosity, whereas microscopy reveals structural features like bacterial structure. Molecular biology, using techniques such as electrophoretic mobility, identifies biofilm chemical features such as surface charge and hydrophobicity, which influence adhesion and mechanical qualities. Additionally, metabolic assays provide information about biofilm activity and strength. Combining these approaches provides a more thorough understanding of biofilm structure, biochemical relationships, and mechanical behavior.

In industrial settings, analytical methodologies for biofilm monitoring must be feasible and scalable. Rheometry is ideal for real-time monitoring because of its capacity to track changes in elasticity, viscosity, and shear behavior, making it useful in industries such as wastewater treatment and food manufacturing. Portable rheometers make it more practical to use. While microscopy, such as CLSM, gives rich visual data, its complexity and setup requirements limit its use in real time. This could be improved with advances in automated imaging. Molecular biology approaches, such as electrophoretic mobility, provide precise insights but are less appropriate for continuous monitoring. Metabolic tests, such as ATP bioluminescence, provide quick input on the activity of microbes, making them excellent for early biofilm detection. A combined approach which employs rheometry for mechanical monitoring and metabolic assays for the viability of biofilm could be beneficial in real-time biofilm control in industries.

## 7. Implications for Food Safety and Industry

### 7.1. Potential Risks Associated With Biofilm Formation

Biofilm formation in the context of food processing and storage is not without its inherent risks, demanding a comprehensive analysis of potential hazards. Gutierrez et al. [159] underscore the fundamental concern of biofilms providing a protective haven for pathogenic bacteria, thereby undermining conventional cleaning and sanitation procedures. The resilience of biofilms to routine disinfection protocols poses a significant risk, potentially resulting in contamination and subsequent foodborne illnesses [160]. Furthermore, the complex microbial communities residing within biofilms raise concerns about the transfer of undesirable microorganisms into final food products, thereby jeopardizing consumer health [161].

Of paramount concern is the ability of biofilms to foster antibiotic resistance among bacteria, a phenomenon that has broader implications for public health [162]. Antibiotic-resistant strains within biofilms could render traditional therapeutic interventions ineffective, exacerbating the challenge of managing bacterial infections. These risks necessitate a thorough exploration of strategies for effective control and prevention.

### 7.2. Strategies for Controlling and Preventing Biofilm Formation

The strategies discussed in this section, their descriptions, applications in the food industry, and relevant references are summarized in Table 3.

Among all these strategies (Table 3), rigorous cleaning should be considered as the most effective method for managing and avoiding biofilm formation in the food sector. Its advantages, such as high effectiveness and options for customization, exceed its disadvantages, especially given the vital requirement to maintain hygienic standards in food processing facilities. Implementing a rigorous cleaning regimen establishes a solid basis for biofilm control; however, other strategies can be used in combination to improve overall effectiveness.

Mitigating the risks associated with biofilm formation commences with the implementation of rigorous cleaning and sanitation protocols. Extensive literature emphasizes the importance of regular and thorough cleaning practices for processing equipment and storage surfaces [163]. The choice of antimicrobial agents and disinfectants plays a pivotal role, and tailoring these agents to specific biofilm-forming microorganisms enhances the efficacy of cleaning procedures [168].

Innovative approaches involve the utilization of bacteriophages and enzymatic disruption to specifically target biofilms. Noteworthy studies have demonstrated the efficacy of phage mixtures, isolated from *Enterobacter cloacae* and *Pseudomonas fluorescens*, in eliminating mature biofilms formed in food matrices [164]. Enzymes, such as glycoside hydrolases and proteases, contribute to the degradation of biofilm exopolysaccharides and the disruption of peripheral proteins [165, 166].

The rheological properties of food matrices significantly influence biofilm formation and removal. The viscosity of food products, for instance, affects the adhesion and detachment of microorganisms [169]. Therefore, strategies for controlling and preventing biofilms must be tailored to the specific rheological attributes of each food product.

Harnessing the potential of natural products adds an eco-friendly dimension to biofilm control strategies. Various compounds, such as carotenoids, garlic oil, and cinnamon essential oil, exhibit antibacterial properties against biofilm-forming microorganisms [167]. The integration of these compounds into food processing practices provides a sustainable approach to biofilm control without compromising the sensory qualities of the final products.

Real-time monitoring and surveillance programs are instrumental in early detection of biofilm formation. Advanced imaging techniques and microbial analysis enable the identification of biofilm-prone areas, facilitating prompt intervention [170]. Proactive management, informed by continuous monitoring, contributes significantly to the mitigation of biofilm-related risks in the food industry.

### 7.3. Consideration of Rheology in Biofilm Control Strategies

The type of fluid that interacts with biofilms also plays a crucial role in determining how effectively cleaning and prevention strategies work. Non-Newtonian fluids provide challenges because of their irregular viscosity and flow characteristics. Shear forces in these fluids are unevenly dispersed across the biofilm surface, which, depending on the flow conditions, can help or inhibit biofilm removal [171]. Designing optimal cleaning solutions and processes thus requires an understanding of the rheological behavior of non-Newtonian fluids, especially in the food industry where non-Newtonian fluids including sauces, syrups, and emulsions are common. The viscosity of certain food matrices has been identified as a critical factor influencing biofilm formation. High viscosity may promote the attachment of microorganisms to surfaces, creating an environment conducive to biofilm development [63]. Therefore, understanding the viscosity of food products is essential for selecting appropriate cleaning agents and designing sanitation procedures.

The application of shear forces during the processing or transportation of food products influences biofilm detachment. Strategies that harness controlled shear forces, coupled with rheologically optimized cleaning solutions, can aid in the effective removal of biofilms [172]. This consideration is particularly relevant in industries where fluid flow and agitation are integral to processing.

Rheologically tailored cleaning approaches involve the development of cleaning agents that match the flow characteristics of specific food products [173]. Customized formulations ensure uniform coverage and enhanced penetration into biofilms, maximizing the effectiveness of cleaning procedures.

## 8. Future Perspectives and Research Directions

Understanding rheology's impact on biofilm formation involves addressing significant knowledge gaps and challenges. The intricate relationship between specific rheological attributes and biofilm architecture needs deeper exploration, especially regarding the synergistic contributions of viscosity and shear forces. Additionally, the role of specific EPS components, beyond alginate, in shaping rheological properties and subsequent biofilm characteristics requires attention. Challenges also lie in deciphering temporal dynamics and the impact of environmental fluctuations on rheological properties and biofilm responses.

Future research must adopt a multidisciplinary approach, integrating microbiology, materials science, and engineering for a holistic understanding of rheology–biofilm interactions. Advanced imaging techniques, like in situ microscopy, can provide real-time insights. Collaborations between microbiologists, rheologists, and engineers can contribute to accurate models. Exploring microbial diversity's influence on biofilm rheology and investigating novel rheological modifiers or biofilm-disrupting agents are promising. Further research on the role of rheology in the formation of biofilm should fill critical knowledge gaps, such as the impact of individual EPS components, microbial diversity, and environmental conditions on biofilm structure. Promising research topics include researching the temporal dynamics of biofilm rheology and investigating rheological modifiers for biofilm control. Advanced techniques include microfluidics-based rheometry for high-resolution shear study, atomic force microscopy (AFM), and multiscale computational modeling to forecast biofilm behavior. Dynamic light scattering, small-angle neutron scattering, and Raman spectroscopy combined with rheometry can improve understanding of biofilm structure and mechanics in different conditions, providing new insights into biofilm–rheology interactions. These developments will not only improve biofilm management but will also influence regulations in areas such as food safety, where rheology can be included into cleaning and preventative measures.

As understanding grows, integrating rheology into food safety regulations becomes imperative. Recognizing rheology as a critical factor in microbial attachment can lead to informed guidelines. Viscosity should be considered in cleaning agent design, and industry standards should incorporate rheological assessments. Interdisciplinary collaborations can formulate standardized protocols, including rheological parameters, for biofilm prevention. Integrating rheology into food safety regulations ensures a proactive approach to biofilm-related risks in the food industry.

## 9. Conclusion

This comprehensive analysis highlights the intricate interplay between rheology and biofilm formation, emphasizing the critical implications for food safety and industry practices. Our findings highlight that biofilms, while providing protection for pathogenic bacteria, present significant issues in food processing and storage due to their resistance to standard cleaning procedures. The various microbial communities seen in biofilms can facilitate the transfer of pathogenic microbes into food products, threatening consumer health and contributing to the development of antibiotic-resistant strains.

This paper methodically details several analytical techniques, such as rheometry and microscopy, that offer insights into biofilm structure and behavior, however with limitations that demand a multifaceted approach for comprehensive assessment. Effective biofilm control strategies are paramount; rigorous cleaning emerges as the most effective method, supplemented by innovative approaches like enzymatic disruption and bacteriophage utilization. These strategies should be tailored to the specific rheological characteristics of food products, acknowledging that the viscosity and flow behavior of non-Newtonian fluids play a crucial role in microbial adhesion and detachment.

Furthermore, incorporating natural products in the control of biofilm provides a sustainable and environmentally friendly alternative, which aligns with the growing desire for safer food processing procedures. The relevance of real-time monitoring and the use of interdisciplinary strategies strengthen our capacity to manage biofilm-related risks. As we move forward, addressing existing knowledge gaps—such as the relationships between rheological properties, EPS components, and biofilm architecture—is essential. Further research should employ advanced methods and collaboration across scientific fields to develop better biofilm management strategies. Recognizing and using rheological principles into food safety regulations allow the industry to better protect public health while also improving overall food safety and quality. Finally, a thorough understanding of the rheology–biofilm dynamics would not only improve biofilm management but also encourage the development of innovative food safety procedures, assuring a healthier future for consumers.

## Figures and Tables

**Table 1:** Impact of rheological modification on biofilms.

**Modification**	**Reference(s)**
Surface coatings: Modifying the surface properties of materials through coatings can influence the attachment of microorganisms. Hydrophobic or hydrophilic coatings can alter the wettability of surfaces, affecting how biofilms adhere.	[114]
Polymer additives: Production of extracellular polymeric substances (EPSs) or introducing polymers to a fluid can alter its rheological properties. For example, adding polymers like guar gum or xanthan gum can increase viscosity. Such modifications can impact the ability of microorganisms to settle and attach to surfaces.	[115]
Surfactants: Surfactants can influence the surface tension of liquids, affecting wetting and spreading. Changes in the surface tension can impact microbial attachment and biofilm initiation. Some surfactants may also disrupt biofilms by affecting their stability.	[114]
Viscosity modifiers: Adding substances that alter the viscosity of a fluid can impact the transport of nutrients and chemicals. For instance, modifying the viscosity with substances like carboxymethyl cellulose can influence the diffusion of molecules through the fluid, affecting biofilm growth.	[114]
Ionic strength adjustments: Changing the ionic strength of a solution can impact its rheological properties. This can be achieved by adjusting the concentration of salts. Ionic strength alterations may influence microbial adhesion and biofilm formation.	[116]
Temperature changes: Variations in temperature can affect the viscosity of liquids. Biofilms exposed to temperature fluctuations may experience changes in their surrounding fluid dynamics, potentially influencing attachment and growth.	[117, 104]
pH modifications: Altering the pH of the environment can influence the charge of surfaces and the solubility of substances. Changes in pH may impact microbial attachment and biofilm development.	[118]
Nutrient availability: Adjusting the composition and concentration of nutrients in a solution can impact the growth and metabolic activity of microorganisms in a biofilm. This, in turn, can influence the overall structure and stability of the biofilm. Biofilm porosity, a crucial determinant of mass transport and nutrient availability, is intricately linked to rheological factors like elastic modulus and viscosity of the biofilm. Extracellular polymeric substance production or rheological modification such as polymer additives potentially induces shifts in biofilm porosity, impacting the diffusion of substances critical for biofilm sustenance. This has significant implications for the design of antibiofilm strategies targeting nutrient deprivation.	[116, 115, 119]

**Table 2:** Summary of analytical techniques for assessing rheology–biofilm interactions.

**Technique**	**Application**	**Principles and methods**	**Best industrial application**	**Potential for real-time monitoring**
Rheometry	Quantifying mechanical properties	Interfacial rheological time sweeps for dynamic biofilm strength, amplitude sweeps for shear behavior, Lissajous plots for structural insights	Pipeline monitoring, water treatment plants, food production lines	High potential for real-time monitoring due to continuous tracking of mechanical properties
Microscopy	Visualizing molecular architecture	CLSM for high-resolution imaging, light microscopy for morphological assessments	Medical device contamination, wastewater biofilm visualization	Low potential due to setup complexity and need for sample prep
Molecular biology approaches	Molecular-level information	Electrophoretic mobility measurements for surface charge and hydrophobicity dynamics	Oil–water separation industries, bioreactors	Low potential, requires controlled conditions for reliable measurements
Metabolic assays	Assessing cell viability and activity	MTT assay and similar tests to evaluate biofilm metabolic activity based on mitochondrial function	Early biofilm detection in cooling towers, water treatment	High potential for real-time monitoring, especially ATP bioluminescence for quick biofilm detection
Metabolite profiling	Analyzing metabolic byproducts	Detection and quantification of biofilm-related metabolites using chromatography or mass spectrometry	Bioprocess monitoring, pharmaceutical production	Medium potential if combined with automated systems, but complex setup limits feasibility for real-time use

**Table 3:** Strategies for controlling and preventing biofilm formation.

**Strategy**	**Description**	**Application in food industry**	**Advantages**	**Disadvantages**	**References**
Rigorous cleaning	Thorough cleaning practices	Processing equipment and storage surfaces	Highly effective in removing biofilms Can be tailored to specific surfaces and contaminants	Labor-intensive and time-consuming May require specialized equipment Effectiveness can be compromised if not performed regularly	[163]
Bacteriophages	Utilization of phage mixtures	Elimination of mature biofilms in food matrices	Highly specific to target bacteria Minimal impact on beneficial microbiota	Regulatory challenges for use in food Potential for phage resistance Production and storage may be complex	[164]
Enzymatic disruption	Use of glycoside hydrolases and proteases	Degradation of biofilm exopolysaccharides	Effective in breaking down biofilm structure Can be used in conjunction with other methods	Possible high costs of enzyme production Complexity of application and stability issues Requires specific conditions for optimal activity	[165, 166]
Natural products	Application of compounds (e.g., carotenoids and garlic oil)	Antibacterial properties against biofilm-forming microorganisms	Eco-friendly and sustainable Generally safe for human consumption Can enhance sensory qualities of food	Variable efficacy depending on concentration May not be as effective against all biofilm-forming organisms Potential for taste alterations	[167]

## Data Availability

The data reviewed for this manuscript can be found in the link: https://drive.google.com/drive/folders/1qc-fgO3XbG5pPhn0nj6XK1PyGVmJBro0?usp=drive_link.
